# Large-Scale Production of Large-Size Atomically Thin Semiconducting Molybdenum Dichalcogenide Sheets in Water and Its Application for Supercapacitor

**DOI:** 10.1038/srep26660

**Published:** 2016-05-26

**Authors:** Yu-Xiang Chen, Chien-Wei Wu, Ting-Yang Kuo, Yu-Lung Chang, Ming-Hsing Jen, I-Wen Peter Chen

**Affiliations:** 1 Department of Applied Science, National Taitung University, 369, Sec. 2, University Rd., Taitung City 95092, Taiwan; 2 Department of Chemistry, National Taiwan University, 1, Sec. 4, Roosevelt Road, Taipei, 10617, Taiwan

## Abstract

To progress from laboratory research to commercial applications, it is necessary to develop an effective method to prepare large quantities and high-quality of the large-size atomically thin molybdenum dichalcogenides (MoS_2_). Aqueous-phase processes provide a viable method for producing thin MoS_2_ sheets using organolithium-assisted exfoliation; unfortunately, this method is hindered by changing pristine semiconducting 2H phase to distorted metallic 1T phase. Recovery of the intrinsic 2H phase typically involves heating of the 1T MoS_2_ sheets on solid substrates at high temperature. This has restricted and hindered the utilization of 2H phase MoS_2_ sheets suspensions. Here, we demonstrate that the synergistic effect of the rigid planar structure and charged nature of organic salt such as imidazole (ImH) can be successfully used to produce atomically thin 2H-MoS_2_ sheets suspension in water. Moreover, lateral size and area of the exfoliated sheet can be up to 50 μm and 1000 μm^2^, respectively. According to the XPS measurements, nearly 100% of the 2H-MoS_2_ sheets was successfully prepared. A composite paper supercapacitor using the exfoliated 2H-MoS_2_ and carbon nanotubes delivered a superior volumetric capacitance of ~410 F/cm^3^. Therefore, the organic salts-assisted liquid-phase exfoliation has great potential for large-scale production of 2H-MoS_2_ suspensions for supercapacitor application.

Layered two-dimensional (2D) materials have drawn tremendous attention after Geim and Novosolov demonstrated that adhesive tape could be utilized to mechanically exfoliate graphite into one-atom-thick layers that can be used in atomically thin electronic devices^1^. Graphene is the most well-known 2D nano-material owing to its superior physical, chemical and mechanical properties, though it has a zero bandgap which restricts its application in energy storage and low-power electronics^2^. Structurally similar to graphene, the lamellar structure of transition metal dichalcogenides (TMDs) includes planes where the atoms of the transition metal and sulfur are covalently bonded and the nearby planes stack upon each other via a weak coupling interaction^3^. Due to their promising electronic and chemical properties, TMDs have attracted considerable attention in research as 2D layered materials. Recently, there has been a focus on atomically thin TMD preparations, most notably molybdenum dichalcogenides (MoS_2_), because they are expected to show superior capacitive properties, which allows for potential applications in electronic devices^4^,^5^. However, the lack of an effective exfoliation method for the large-scale production of high-concentration atomically thin 2H-MoS_2_ sheets has been one of the obstacles to studying their chemical and physical properties and to using them for novel and practical applications.

Owing to high production prices and limited scalability, mechanical exfoliation limits the use of 2H-MoS_2_ materials to fundamental research and special applications^6^. Similarly, the chemical vapor deposition method may be an alternative route for the scalable production of 2H-MoS_2_ materials. However, it is not only hard to precisely control the stoichiometry ratio, but this method also involves high prices and rather low yields^6^. In contrast, the liquid phase exfoliation method is characterized by high scalability and relatively low production costs, and it does not require transfer techniques, thus making it suitable for producing 2H-MoS_2_ materials in bulk quantities^6^,^7^. Recently, liquid-phase routes have been demonstrated by sonicating MoS_2_ powder in properly chosen solvents such as a mixture of ethanol and water or dimethylformamide and N-methylpyrrolidone, which can directly exfoliate MoS_2_ powder and disperse the exfoliated MoS_2_ sheets owing to their compatible surface energies^8^,^9^,^10^. Coleman *et al.* have demonstrated that liquid-phase exfoliation of MoS_2_ powder in N-methylpyrrolidone with ultrasonication is a practical route to achieving the preparation of MoS_2_ sheets. However, this method yields multi-layered sheets of MoS_2_, and thin sheets are very rarely observed^10^,^11^. Recently, large amounts of multi-layered MoS_2_ suspension were successfully prepared after more than 100 hours of sonication^10^ or several hours of grinding^12^. This, however, provided exfoliated MoS_2_ nanosheets less than 100 nm in size and affected its unique and original properties. Moreover, N-methylpyrrolidone is expensive and requires special attention while handling^13^. Unfortunately, one of the most ideal dispersion media, water, has a non-compatible surface energy that is too high to have an effect on its own for MoS_2_ exfoliation. The best-known group of exfoliants involves electron donors, for example, organolithium compounds^14^,^15^,^16^,^17^,^18^. Bulk MoS_2_ can be exfoliated with lithium (Li) under rigorous conditions for more than 72 hours to obtain reduced Li_x_MoS_2_ with an expanded structure; this can be further exfoliated by a sonication-assisted process^16^,^17^. However, Li-assisted exfoliation leads to a structural change in MoS_2_ from the thermodynamically stable semiconducting 2H phase to the metastable metallic 1T phase^16^,^17^. Moreover, 1T-MoS_2_ turns into nanometer-sized flakes after exfoliation along with the occurrence of metal nanoparticle formation and Li_2_S agglomeration^19^. This has dramatically hindered the development of atomically thin MoS_2_ sheets in most applications that require a pristine structure and large sheets with high concentrations. Recently, an electrochemical method was successfully established to produce MoS_2_ sheets. Nevertheless, mass production through this process is limited because Mo^5+^ and Mo^6+^ are generated^20^,^21^. Exploration has recently been further extended to produce exfoliated MoS_2_ sheets by the use of exfoliants in water or polymers in tetrahydrofuran^22^,^23^. Polymer-assisted exfoliation methods in an aqueous solution with high-power probe sonication were reported, but the final concentration of less than 0.5 mg/mL and resulting small sheets were still far from the demands of practical applications requiring large quantities^22^. Inspired by the liquid phase exfoliation of graphene^24^,^25^,^26^, a method with similar exfoliation principles may be an approach to boost the production of atomically thin 2H-MoS_2_ sheets.

This study demonstrates that atomically thin MoS_2_ sheets in the 2H phase can be prepared by organic salts such as imidazole (ImH) or pyridiniumtribromide (PyBr_3_). The exfoliated 2H-MoS_2_ sheet dimensions can be as large as to 1000 μm^2^. In addition, nearly 100% of the composition was of the thermodynamically stable 2H phase structure. These exfoliants are cheap and commercially available, and the method can be performed under ambient conditions. Moreover, the 2H-MoS_2_ suspension allows for the preparation of high electrical conductive composites for energy storage applications.

## Results and Discussion

**Figure 1:**
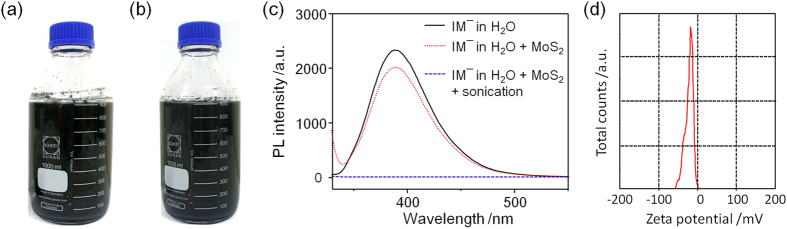
Large-scale production of 2H-MoS_2_ sheets by ultrasonication. (**a**) An Im^−^-assisted exfoliated 2H-MoS_2_ suspension on the liter-scale made by ultrasonic exfoliation. (**b**) The as-prepared 2H-MoS_2_ suspension after standing for more than a year. (**c**) The PL spectra of Im^−^ (black line), Im^−^/2H-MoS_2_ without sonication (red dotted line) and Im^−^/2H-MoS_2_ with sonication (blue dashed line). (**d**) Zeta potential distributions of the Im^−^‒assisted 2H-MoS_2_ suspension.

Figure S1a shows a photograph of the experimental setup for the ultrasonication exfoliation of 2H-MoS_2_ powder (Fig. S1b). A suspension of the exfoliated 2H-MoS_2_ sheets was prepared from commercial 2H-MoS_2_ powder via an ImH or PyBr_3_ exfoliation process followed by ultrasonication. Figure 1a shows that liter-scale 2H-MoS_2_ suspensions can be prepared through Im^−^-assisted exfoliation. These highly-dispersed suspensions of 2H-MoS_2_ sheets can stand for more than a year, and the suspension remained dark green in color, as shown in Fig. 1b. To unveil the exfoliation mechanism of the layered 2H-MoS_2_, we performed a photoluminescence (PL) experiment. First, the Im^−^ solution showed PL emission at 388 nm (Fig. 1c; black solid line) under irradiation at 270 nm. Then, the MoS_2_ powder was added to the Im^−^ solution. After shaking the mixture, the 2H-MoS_2_/Im^−^ solution was left on the bench for 30 minutes (Fig. 1c; red dotted line). The PL intensity of the 2H-MoS_2_/Im^−^ solution decreased, indicating that Im^−^ molecules, nearly one atom in thickness, adsorbed to the layered 2H-MoS_2_. Finally, the MoS_2_ powder was exfoliated by ultrasonication to generate large quantities of the atomically thin 2H-MoS_2_ sheets in suspension. Interestingly, the PL intensity of 2H-MoS_2_/Im^−^ was fully quenched (Fig. 1c; blue line), which demonstrates that nearly all of the Im^−^ molecules adsorbed to the exfoliated 2H-MoS_2_ surfaces. The zeta potential of the suspension of the exfoliated 2H-MoS_2_ sheets was −22.4 mV (Fig. 1d), which demonstrated its superior aqueous stability with the assistance of Im^−^ molecules. The negative surface charges provide evidence of electrostatic repulsion forces between each of the exfoliated 2H-MoS_2_ sheets, resulting in a stable aqueous suspension. In the X-ray diffraction (XRD) patterns, the intensity of the (002) reflection of the exfoliated 2H-MoS_2_ is significantly weaker than the bulk MoS_2_ powder, which means that a large amount of the exfoliated 2H-MoS_2_ thin sheets prepared were highly exfoliated (Fig. S2)^21^,^27^,^28^,^29^. Just as reported in the literature, the planar structure of the MoS_2_ flakes was hydrophobic^30^,^31^. Meanwhile, the ring of the Im^−^ molecules also possesses a hydrophobic property^32^. Therefore, the mechanism for the Im^−^-assisted exfoliation of layered 2H-MoS_2_ materials is attributed to hydrophobic-hydrophobic interaction. The exfoliated 2H-MoS_2_ flakes are stabilized due to the electrostatic repulsive forces generated by the planar charged organic molecules.

**Figure 2:**
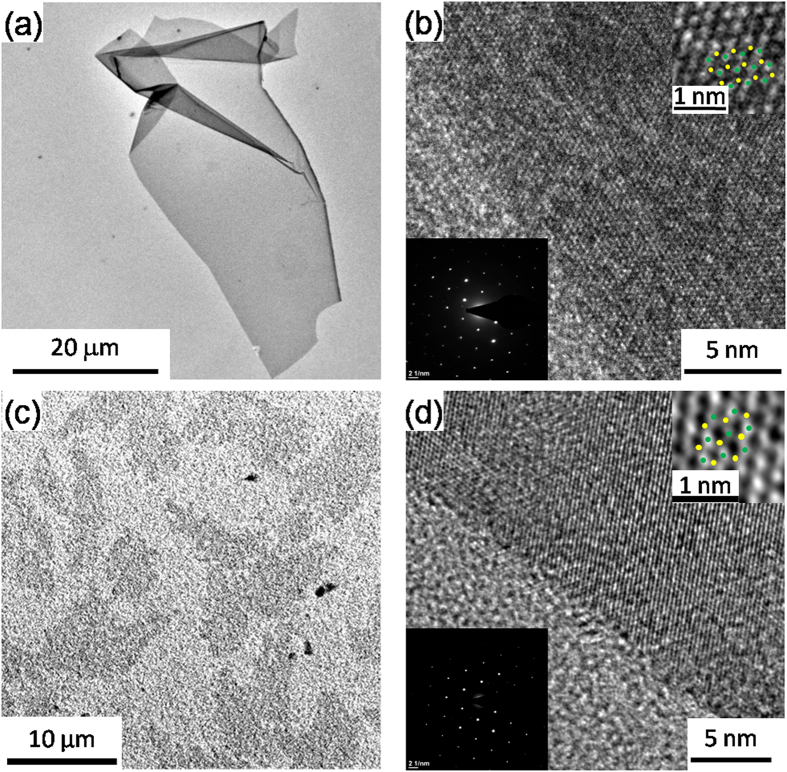
Morphological characterization of the exfoliated 2H-MoS_2_ sheets. (**a**,**c**) TEM images of Py^+^‒assisted and Im^−^‒assisted exfoliated 2H-MoS_2_ sheets, respectively. (**b,d**) HRTEM images of Py^+^‒assisted and Im^−^‒assisted exfoliated 2H-MoS_2_ sheets, respectively. The bottom insets of (**b,d**) show the selected area electron diffraction (SAED) pattern of the Py^+^‒assisted and Im^−^‒assisted exfoliated 2H-MoS_2_ sheets, respectively. Upper insets show individual Mo (green dot) and S (yellow dot) atoms and their honeycomb arrangement.

Figures 2a,c show transmission electron microscopy (TEM) images of a thin 2H-MoS_2_ sheet. Figure S3 shows large flakes of the exfoliated 2H-MoS_2_. The exfoliated 2H-MoS_2_ sheets are characterized through the edge of the sheets which shows that large-area 2H-MoS_2_ flakes are mostly all monolayer via the high resolution TEM (HRTEM) images as shown in the inset of Fig. S3. The exfoliated 2H-MoS_2_ sheets had a maximum lateral size of over 50 μm, which is nearly two orders of magnitude larger than using exfoliant-assisted methods^16^,^17^ or the solvent exfoliated method^11^,^12^. Intriguingly, the production of thin 2H-MoS_2_ sheets with an unprecedented sheet size of up to 1000 μm^2^ was achievable (Fig. 2a). The HRTEM images in Fig. 2b,d show that the lattice structure of the edge of the exfoliated 2H-MoS_2_ sheet was not damaged during the ultrasonic exfoliation process. In addition, the diffraction patterns of the insets in Fig. 2b,d show lattice spacing of 0.2764 and 0.2797 nm, respectively; both match the (100) plane of typical 2H-MoS_2_ sheets^33^. On the basis of the above results, the synergistic effect of the charged nature and the planar structure of the Im^−^ and Py^+^ enabled both the exfoliation of 2H-MoS_2_ and the stabilization of the 2H-MoS_2_ suspension. Besides that, atomic force microscopy (AFM) is a powerful tool used in assessing the thickness of exfoliated 2H-MoS_2_ sheets, which in turn correlates to the number of layers. The average thickness of 743 measured sheets from the substrate to the sheet was found to be ~0.9 nm, as shown in Fig. S4a. This statistical thickness is in good agreement with the reported 2H-MoS_2_ monolayer thickness of 0.9 ~ 1.2 nm^11^,^16^. Figure S4b indicates that the flake size of most of the exfoliated 2H-MoS_2_ sheets ranged from a few square micrometers to one thousand square micrometers. Based on the results of the PL, zeta potential, XRD, TEM and AFM measurements, a proposed schematic illustration of 2H-MoS_2_ exfoliation is shown in Fig. S5.

**Figure 3:**
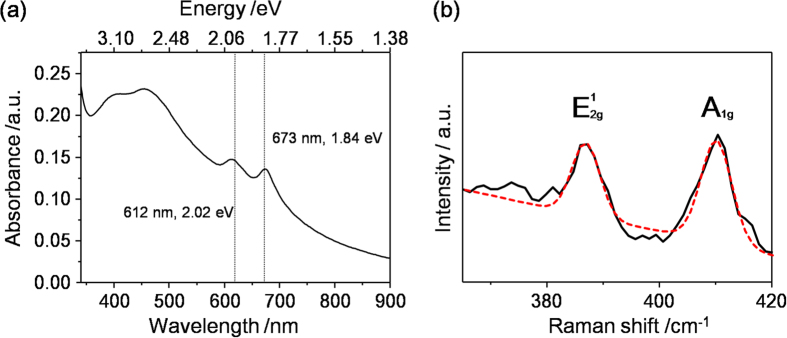
(**a**) UV-vis absorption and (**b**) Typical Raman spectrum of Im^−^‒assisted exfoliated 2H-MoS_2_ sheets. (black line: experimental data; red dotted line: fitted peaks).

Further structural characterization of the already-prepared concentration of the exfoliated 2H-MoS_2_ sheets (1 mL) was diluted with about 400 mL of water and used to evaluate the absorption spectrum of the 2H-MoS_2_ sample, as shown in Fig. 3a. The peaks centered at 400, 451, 612 and 673 nm were the representative absorption bands of the exfoliated 2H-MoS_2_ in solution. The peaks at 400 and 451 nm could be ascribed to the direct transition from the valence band to the conduction band. The excitonic peaks at 612 and 673 nm, responsible for the K point of the Brillouin zone, were also clearly observed. These two characteristic peaks demonstrate the existence of high-quality atomically thin 2H-MoS_2_ sheets. By utilizing Beer’s law and the extinction coefficient centered at 672 nm α_672_ = 3400 mL/(mg·m)^11^,^34^, we can estimate the concentration of the exfoliated MoS_2_ sheets was ~2 mg/mL in our supernatant. However, the extinction coefficient may change with the solvent, so the value of 2 mg/mL should be considered a rough estimate^34^. To obtain a more accurate concentration, we filtrated the already-prepared 2H-MoS_2_ suspension. Then, it was dried at 200 ^o^C for 2 h to remove the physisorbed exfoliant of Im^−^. The concentration of the exfoliated 2H-MoS_2_ suspension was ~4 mg/mL. The Im^−^-assisted exfoliated 2H-MoS_2_ sheets exhibited two Raman characteristic peaks at around 387 and 409 cm^−1^ with full-width-half-maximum (FWHM) values of 3.6 and 6.8 cm^−1^, corresponding to the E^1^_2g_ and A_1g_ modes, respectively, as shown in Fig. 3b. Note that the Raman frequency difference between E^1^_2g_ and A_1g_ is consistent with that of chemically exfoliated single-layer MoS_2_^35^. The ratio (E^1^_2g_/A_1g_) of the integrated intensity of the Im^−^-assisted exfoliated 2H-MoS_2_ sheets was 0.59. Moreover, Fig. S6 shows that the Py^+^-assisted exfoliated 2H-MoS_2_ also showed two Raman characteristic peaks with FWHM values of 6.2 and 4.4 cm^−1^, corresponding to A_1g_ and E^1^_2g_ modes, respectively. The ratio (E^1^_2g_/A_1g_) of the integrated intensity of the Py^+^-assisted exfoliated MoS_2_ sheet was 0.47. The peak FWHM values and the integrated intensity ratios were similar to those reported after mechanical exfoliation of single-layer 2H-MoS_2_ sheets, demonstrating the successful preparation of single-layer 2H-MoS_2_ sheets^36^.

**Figure 4:**
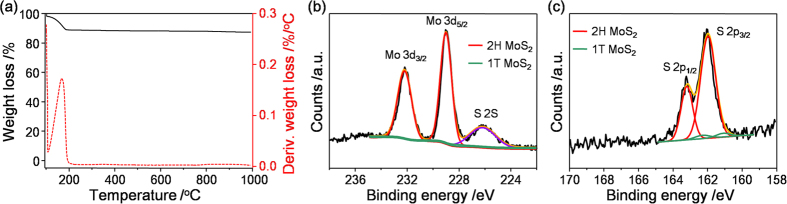
(**a**) TGA test of the exfoliated 2H-MoS_2_ sheets (black line); differential weight loss (%*/*d*T*, red dashed lines) is shown as a function of temperature. (**b**) XPS spectrum showing the peak regions of Mo 3d and S 2s core level for the exfoliated 2H-MoS_2_ sheets. (**c**) S 2p core level of the XPS spectrum for the exfoliated 2H-MoS_2_ sheets. The samples were calibrated using the Au 4f_5/2_ line at 84.0 eV as a reference. After Shirley background subtraction, the peaks were fitted with Gaussian curve fitting. The Mo 3d and S 2p were deconvoluted to exhibit the 2H and 1T contributions, shown as red and green lines, respectively.

We employed thermogravimetric analysis (TGA) to explore the thermal stability of the exfoliated 2H-MoS_2_ sample. Figure 4a shows the TGA profile of the Im^−^-assisted exfoliated 2H-MoS_2_ sheets. The TGA curve of the exfoliated 2H-MoS_2_ sheets shows one weight loss stage around 160 ^o^C. The weight loss region with about 12% loss of the original weight which occurred around 110–190 ^o^C was due to the decomposition of the adsorbed imidazolium molecules. After 200 ^o^C, no further weight loss occurred. The differential TGA curve showed the Im^−^-assisted exfoliated MoS_2_ sheets had one major peak at 167 ^o^C, which is consistent with the decomposition temperature of pure imidazole (Fig. S7). The TGA result demonstrates that there was no detectable oxidization in the exfoliated 2H-MoS_2_ sheets. This confirmed that the production process did not result in the formation of S–O derivatives on the exfoliated 2H-MoS_2_ sheets. According to previous studies, the thermodynamically stable 2H phase of MoS_2_ is the trigonal prismatic phase where every molybdenum atom is coordinated by six neighboring sulfur atoms^16^. However, the MoS_2_ structure could form a metastable phase where the coordination of Mo atoms becomes octahedral (1T-MoS_2_) upon organolithium-assisted exfoliation^14^,^16^,^17^,^37^. Therefore, we studied the phase composition of the exfoliated MoS_2_ sheets with X-ray photoelectron spectroscopy (XPS). The Mo 3d of the XPS spectrum is shown in Fig. 4b. The Mo 3d spectrum shows peaks centered at 229.1 and 232.3 eV, corresponding to the Mo^4+^ 3d_5/2_ and Mo^4+^ 3d_3/2_ components of 2H-MoS_2_, respectively. In addition, the spectrum shows two unobservable peaks at 228.3 and 231.4 eV, corresponding to the Mo^4+^ 3d_5/2_ and Mo^4+^ 3d_3/2_ components of 1T-MoS_2_, respectively. Similarly, in the S 2p core level spectrum, Fig. 4c shows that two peaks were observed at 162.0 and 163.3 eV, corresponding to the S 2p_3/2_ and S 2p_1/2_ components of 2H-MoS_2_, respectively. Additionally, the spectrum shows two weak peaks at 161.1 and 162.4 eV, corresponding to the S 2p_3/2_ and S 2p_1/2_ components of 1T-MoS_2_, respectively. Moreover, no signals were detected in the range of 166 to 170 eV, indicating that sulfur elements also persisted in non-oxidized form. These results are in agreement with reported studies on MoS_2_ single crystals, showing that the exfoliated MoS_2_ sheets are predominantly in the 2H phase^16^. The exfoliated MoS_2_ sheets contain over 95% of the 2H phase after exfoliant-assisted exfoliation. The remaining less than 5% of the non-2H phase was nearly fully converted to the 2H phase after annealing at 200 ^o^C (Fig. S8). This exfoliation method demonstrates that exfoliated 2H-MoS_2_ sheets preserve their thermodynamically stable semiconducting properties. This is in contrast to Li exfoliated MoS_2_ in which the semiconducting properties of the sheets are perturbed due to their phase structural changes from the 2H to 1T phase. In addition, it is worth mentioning that the peak at 236 eV did not show a significant signal, attributed to Mo^6+^ 3d_5/2_, indicating that the oxidized form of Mo^6+^ is difficult to detect. This result is superior to that of electrochemically exfoliated MoS_2_ sheets that oxidize the pristine charge of Mo^4+^ to Mo^5+^ and Mo^6+ ^21.

**Figure 5:**
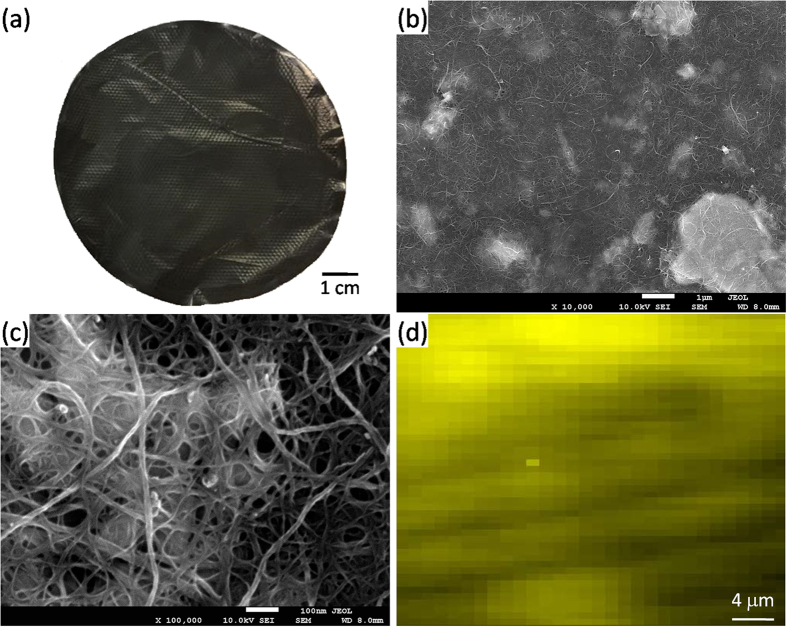
2H-MoS_2_/CNTs composite paper. (**a**) Photograph of the free-standing hybrid paper of 2H-MoS_2_/CNTs. (**b**) low and (**c**) high magnification of FE-SEM images of the 2H-MoS_2_/CNTs hybrid paper. (**d**) Raman image of the hybridized paper by extracting the frequency of the characteristic peak of E^1^_2g_. The Raman 2D mapping area is 40 × 60 μm.

The versatility of the exfoliation method allowed us to fabricate composite papers by directly adding dispersed carbon nanotubes (CNTs) to the 2H-MoS_2_ suspension. Free-standing 2H-MoS_2_/CNTs papers were peeled off from the filtered membrane, as shown in Fig. 5a. Scanning electron micrographs (SEM) of the 2H-MoS_2_/CNTs paper at low (Fig. 5b) and high (Fig. 5c) magnification clearly showed that the 2H-MoS_2_ sheets were wrapped in CNTs. To clearly understand the distribution of the 2H-MoS_2_ sheets in the 2H-MoS_2_/CNTs paper, Fig. 5d shows the mapping image from Raman spectroscopy of the composite paper by extracting the frequency of the characteristic peak of E^1^_2g_. The information in the Raman mapping spectrum shows similar Raman intensity of E^1^_2g_, which indicates a uniform dispersion of the 2H-MoS_2_ in the composite paper. To compare the electrical conductivity of the composite papers, we compared a pure MoS_2_ crystal with the composite papers. The addition of CNTs increased the electrical conductivity, σ, from ~10^−6^ S/cm for the 2H-MoS_2_ only film to ~6 × 10^2^ S/cm for the 2H-MoS_2_/CNTs composite paper. The electrical conductivity of the 2H-MoS_2_/CNTs hybrid paper compares superiorly with some of the other 2D materials’ electrodes for supercapacitors^38^. The capacitance of the 2H-MoS_2_/CNTs hybrid paper was obtained using cyclic voltammetry (CV). The resulting CVs for potential ranged from 0 to 0.8 V versus NHE, as shown in Fig. S9a. The galvanostatic charging/discharging curves for the 2H-MoS_2_/CNTs paper were recorded at different current densities as depicted in Fig. S9b. Figure S9c shows the volumetric capacitances at various current densities of supercapacitors based on 2H-MoS_2_/CNT composite papers. The volumetric capacitance of the 2H-MoS_2_/CNT hybridized supercapacitor was ~410 F/cm^3^ at a current density of 2 A/cm^3^. This value is superior to that of a film-based supercapacitor consisting of ZnO/graphene film (0.36 F/cm^3^)^39^ MoS_2_/CNTs (0.7 F/cm^3^)^40^, MoS_2_/reduced graphene oxide/CNTs (5.2 F/cm^3^)^40^, and reduced graphene oxide/MoS_2_ (30 F/cm^3^)^41^. Moreover, it is higher than the value of the best micro-supercapacitor based on carbide-derived carbon film^42^. The improved performance of the 2H-MoS_2_/CNTs paper electrodes could be possibly attributed to three reasons. First, the Mo atoms of the exfoliated 2H-MoS_2_ sheets possess many oxidation states from +2 to +6, promising a representative pseudo-capacitance behavior with high specific capacitance for 2H-MoS_2_/CNTs hybrid paper^43^. Second, the interconnected high conducting CNTs network provides electric double-layer capacitance and also serves as a conductive material for charge transport and transfer^42^,^43^. Finally, the dispersed CNTs of the 2H-MoS_2_/CNTs composite paper serve as spacers to mitigate restacking between the exfoliated 2H-MoS_2_ sheets^44^,^45^. The cyclic stability of the 2H-MoS_2_/CNTs hybrid paper electrodes in a 0.5 M K_2_SO_4_ electrolyte was tested over 2,300 cycles (Fig. S9d) and maintained a capacitance in excess of 95% after 2,300 cycles.

## Conclusion

We have demonstrated a universal route to the large-scale production of a high-concentration of large, atomically thin 2H-MoS_2_ sheets that can be made by adding an organic salt. The lateral size and area of the exfoliated 2H-MoS_2_ can reach 50 μm and 1000 μm^2^, respectively, which is nearly two orders of magnitude greater than that of mechanically, Li-assisted chemically exfoliated MoS_2_ sheets. The spectral analysis, which incorporated TEM, AFM, XRD, UV-vis, PL, Raman, XPS and TGA measurements, confirmed that the exfoliated MoS_2_ sheets predominately exhibited the 2H phase. We anticipate that fast progress in areas like hybrid paper processing and electronic device fabrication involving large-scale production of high-concentration, large, atomically thin 2H-MoS_2_ sheets will develop from these results.

## Methods

### Materials

Molybdenum (IV) sulfide (99% metals basis; ~325 mesh powder) were purchased from Alfa Aesar. Imidazole (99%, ACROS) and pyridinium tribromide (TCI) were used without further purification as the exfoliant. Considering large-scale production of the 2H-MoS_2_ suspension and the high cost, ImH is preferred. Single-walled carbon nanotubes powder (CG300-L16) were produced by SouthWest NanoTechnologies Inc. Multi-walled carbon nanotubes powder and Triton X-100 utilized in this study were produced by Golden Innovation Business Co. Ltd.

### Exfoliation of MoS_2_ powder

To exfoliate MoS_2_ powder to form 2H-MoS_2_ suspension, a high concentration of exfoliated 2H-MoS_2_ sheets was prepared by using a Chromtech model UP-500 ultrasonic homogenizer with a ½-inch ultrasonic tip. 10 g of the MoS_2_ powder, 20 g of imidazole and 1000 mL of deionized water (Elga Ltd., HighWycombe, Bucks, UK) was added to a 1000 mL beaker and sonicated with tip sonication (100 ~ 500 W) in continuous mode for 2 h. The exfoliation process was performed under an N_2_ environment at controlled temperature of 15 ^o^C (CC-1010, Panchun Sci. Corp.).

### Preparation of 2H-MoS_2_/CNT composite papers

The 108 mg CNT was sonicated in a water bath with the assistance of aqueous Triton X-100 dispersant for 1 h to achieve the CNT suspension of 1L. Then was poured CNT dispersion of 250 mL and the Im^−^-assisted exfoliated as-exfoliated 2H-MoS_2_ nanosheets suspension of 50 mL into the beaker. The mixture was sonicated with tip sonication (100 W) for 30 minutes to enhance materials mixing. The mixture was filtrated via a filtration membrane with a pore size of 0.2 μm (cellulose acetate, Advantec) under positive pressure. Randomly dispersed 2H-MoS_2_/CNT composite papers with a thickness of ~10 μm were washed thoroughly with distilled water to remove the dispersant and the exfoliant. All 2H-MoS_2_/CNT composite papers were dried at 200 ^o^C under vacuum for 1h to evaporate the moisture, residual dispersant and the exfoliant. producing a final 2H-MoS_2_/CNT composite papers with a electrical conductivity of ~600 S/cm.

### Characterization

The exfoliated 2H-MoS_2_ sheets were deposited by dip coating onto a freshly mica or Si substrate for Raman, PL, AFM and XPS studies. Raman spectra were recorded using a multipurpose spectrometer (iHR550, Horiba Jobin Yvon) with a 532 nm excitation laser source in air under ambient conditions, the power of the laser was set below 100 mW, the spot-size of the laser was approximately 5 μm, and the peak of Si at 520.7 cm^−1^ was used for calibration. The PL spectrum was measured using a PL spectrophotometer (Hitachi F-4500, Japan). The ultraviolet-visible (UV-vis) absorbance spectra were recorded on a Unicam UV-300 UV-vis spectrophotometer using quartz cuvettes. The XPS measurements were carried out by using an Thermo K-Alpha (VGS) with Al Kα X-ray (1486.6 eV) as radiation source. The binding energies were calibrated with Au 4f_7/2_ at 84.0 eV. The morphology and structure of the exfoliated 2H-MoS_2_ sheets were investigated using TEM (JEOL JEM-2100) and HRTEM (Hitachi H-7100) with the exfoliated 2H-MoS_2_ sheets directly transferred onto a formvar-coated or lacey-coated copper grid. A tapping mode atomic force microscope (TM-AFM; Innova/Bruker, Santa Barbara, CA) was used to characterize the thickness of the exfoliated 2H-MoS_2_ sheets. The analyses of sample weight loss were conducted using a TGA instrument (Q500, TA instrument). Electrochemical studies were measured using a CHI 7279E (CH instrument Corp.). The electrical contacts for the 2H-MoS_2_/CNT paper were connected using copper alligator clip. The MoS_2_/CNT paper electrodes of 1 cm × 0.5 cm were tested in 0.5 M Na_2_SO_4_ solution using a typical three-electrode electrochemical system, with a Ag/AgCl electrode as the reference electrode and a Pt wire as the counter electrode. Cyclic voltammetry data were recorded in between 0 V and 0.8 V vs. NHE with scan rate ranging from 1 mV/s up to 300 mV/s. The electrical conductivity of the 2H-MoS_2_/CNT papers was recorded using the four-point probe method with a probe diameter of 80 μm and a distance of 1.6 mm between two adjacent probes. Measurements were carried out using a KeithLink probe station in order to provide current between −5 mA to 5 mA. Each sheet of 2H-MoS_2_/CNT paper was cut into 5 cm × 5 cm squares for the electrical measurements.

## Additional Information

**How to cite this article**: Chen, Y.-X. *et al.* Large-Scale Production of Large-Size Atomically Thin Semiconducting Molybdenum Dichalcogenide Sheets in Water and Its Application for Supercapacitor. *Sci. Rep.* **6**, 26660; doi: 10.1038/srep26660 (2016).

## Supplementary Material

Supplementary Information

## References

[b1] Novoselov K. S. *et al.* Electric Field Effect in Atomically Thin Carbon Films. Science 306, 666–669 (2004). 15499015 10.1126/science.1102896

[b2] Schwierz F. Graphene Transistors: Status, Prospects, and Problems. Proc. IEEE 101, 1567–1584 (2013).

[b3] Novoselov K. S. *et al.* Two-Dimensional Atomic Crystals. Proc. Natl. Acad. Sci. USA 102, 10451–10453 (2005). 16027370 10.1073/pnas.0502848102 PMC1180777

[b4] Das S. *et al.* Beyond Graphene: Progress in Novel Two-Dimensional Materials and van der Waals Solids. Annu. Rev. Mater. Res. 45, 20.21–20.27 (2015).

[b5] Butler S. Z. *et al.* Progress, Challenges, and Opportunities in Two-Dimensional Materials Beyond Graphene. ACS Nano 7, 2898–2926 (2013). 23464873 10.1021/nn400280c

[b6] Raccichini R., Varzi A., Passerini S. & Scrosati B. The Role of Graphene for Electrochemical Energy Storage. Nat. Mater. 14, 271–279 (2015). 25532074 10.1038/nmat4170

[b7] Tour J. M. Scaling up Exfoliation. Nat. Mater. 13, 545–546 (2014). 24747779 10.1038/nmat3961

[b8] Zhou K.-G. *et al.* A Mixed-Solvent Strategy for Efficient Exfoliation of Inorganic Graphene Analogues. Angew. Chem. Int. Ed. 50, 10839–10842 (2011). 10.1002/anie.201105364 21954121

[b9] Nicolosi V. *et al.* Liquid Exfoliation of Layered Materials. *Science* 340, 1226419 (2013).

[b10] O’Neill A., Khan U. & Coleman J. N. Preparation of High Concentration Dispersions of Exfoliated MoS_2_ with Increased Flake Size. Chem. Mater. 24, 2414–2421 (2012).

[b11] Coleman J. N. *et al.* Two-Dimensional Nanosheets Produced by Liquid Exfoliation of Layered Materials. *Science* 331, 568–571 (2011). 21292974 10.1126/science.1194975

[b12] Yao Y. *et al.* High-Concentration Aqueous Dispersions of MoS_2_. Adv. Funct. Mater. 23, 3577–3583 (2013).

[b13] Lotya M. *et al.* Liquid Phase Production of Graphene by Exfoliation of Graphite in Surfactant/Water Solutions. J. Am.Chem. Soc. 131, 3611–3620 (2009). 19227978 10.1021/ja807449u

[b14] Py M. A. & Haering R. R. Structural Destabilization Induced by Lithium Intercalation in MoS_2_ and Related Compounds. Can. J. Phys. 61, 76–84 (1983).

[b15] Joensen P., Frindt R. F. & Morrison S. R. Single-Layer MoS_2_. Mater. Res. Bull. 21, 457–461 (1986).

[b16] Eda G. *et al.* Photoluminescence from Chemically Exfoliated MoS_2_. Nano Lett. 11, 5111–5116 (2011). 22035145 10.1021/nl201874w

[b17] Zheng J. *et al.* High Yield Exfoliation of Two-Dimensional Chalcogenides using Sodium Naphthalenide. Nat. Commun. 5, 3995 (2014). 24384979 10.1038/ncomms3995

[b18] Wang Q. H. *et al.* Electronics and Optoelectronics of Two-Dimensional Transition Metal Dichalcogenides. Nat. Nanotechnol. 7, 699–712 (2012). 23132225 10.1038/nnano.2012.193

[b19] Chhowalla M. *et al.* The Chemistry of Two-Dimensional Layered Transition Metal Dichalcogenide Nanosheets. Nat. Chem. 5, 263–275 (2013). 23511414 10.1038/nchem.1589

[b20] Zeng Z. *et al.* Single-Layer Semiconducting Nanosheets: High-Yield Preparation and Device Fabrication. Angew. Chem. Int. Ed. 50, 11093–11097 (2011). 10.1002/anie.201106004 22021163

[b21] Liu N. *et al.* Large-Area Atomically Thin MoS_2_ Nanosheets Prepared Using Electrochemical Exfoliation. ACS Nano 8, 6902–6910 (2014). 24937086 10.1021/nn5016242

[b22] Smith R. J. *et al.* Large-Scale Exfoliation of Inorganic Layered Compounds in Aqueous Surfactant Solutions. Adv. Mater. 23, 3944–3948 (2011). 21796689 10.1002/adma.201102584

[b23] May P., Khan U., Hughes J. M. & Coleman J. N. Role of Solubility Parameters in Understanding the Steric Stabilization of Exfoliated Two-Dimensional Nanosheets by Adsorbed Polymers. J. Phy. Chem. C 116, 11393–11400 (2012).

[b24] Ciesielski A. & Samorı P. Graphene via Sonication Assisted Liquid-Phase Exfoliation. Chem. Soc. Rev. 43, 381–398 (2014). 24002478 10.1039/c3cs60217f

[b25] Chen I.-W. P., Jhou S.-H. S. & Chen Y.-W. Preparation of High-Quality Graphene Sheets and Their Applications in Highly Conductive Papers and a High-Performance Electromechanical Actuator J. Mater. Chem. C 1, 5970–5975 (2013).

[b26] Chen I.-W. P., Huang C.-Y., Jhou S.-H. S. & Zhang Y.-W. Exfoliation and Performance Properties of Non-Oxidized Graphene in Water. Sci. Rep. 4, 3928 (2014). 24473336 10.1038/srep03928 PMC3905268

[b27] Liang Y. *et al.* Rechargeable Mg Batteries with Graphene-like MoS_2_ Cathode and Ultrasmall Mg Nanoparticle Anode. Adv. Mater. 23, 640–643 (2011). 21274912 10.1002/adma.201003560

[b28] Kim C. *et al.* Performances of Liquid-Exfoliated Transition Metal Dichalcogenides as Hole Injection Layers in Organic Light-Emitting Diodes. Adv. Funct. Mater. 25, 4512–4519 (2015).

[b29] Anbazhagan R., Wang H.-J., Tsai H.-C. & Jeng R.-J. Highly Concentrated MoS_2_ Nanosheets in Water Achieved by Thioglycolic Acid as Stabilizer and Used as Biomarkers. Rsc. Adv. 4, 42936–42941 (2014).

[b30] Li Y. *et al.* Nanocellulose as Green Dispersant for Two-Dimensional Energy materials. Nano Energy 13, 346–354 (2015).

[b31] Chow P. K. *et al.* Wetting of Mono and Few-Layered WS_2_ and MoS_2_ Films Supported on Si/SiO_2_ Substrates. ACS Nano 9, 3023–3031 (2015). 25752871 10.1021/nn5072073

[b32] Mi H., Jiang Z. & Kong J. Hydrophobic Poly (ionic liquid) for Highly Effective Separation of Methyl Blue and Chromium Ions from Water. Polymer 5, 1203–1214 (2013).

[b33] Lee Y.-H. *et al.* Synthesis of Large-Area MoS_2_ Atomic Layers with Chemical Vapor Deposition. Adv. Mater. 24, 2320–2325 (2012). 22467187 10.1002/adma.201104798

[b34] Li J. *et al.* Inkjet Printing of MoS_2_. Adv. Funct. Mater. 24, 6524–6531 (2014).

[b35] Voiry D. *et al.* Covalent Functionalization of Monolayered Transition Metal Dichalcogenides by Phase Engineering. Nat. Chem. 7, 45–49 (2015). 25515889 10.1038/nchem.2108

[b36] Lee C. *et al.* Anomalous Lattice Vibrations of Single and Few-Layer MoS_2_. ACS Nano 5, 2695–2700 (2010). 20392077 10.1021/nn1003937

[b37] Imanishi N., Toyoda M., Takeda Y. & Yamamoto O. Study on Lithium Intercalation into MoS_2_. Solid State Ionics 58, 333–338 (1992).

[b38] Zhu Y. *et al.* Graphene and Graphene Oxide: Synthesis, Properties, and Applications. Adv. Mater. 22, 3906–3924 (2010). 20706983 10.1002/adma.201001068

[b39] Bae J. *et al.* Single-Fiber-Based Hybridization of Energy Converters and Storage Units using Graphene as Electrodes. Adv. Mater. 23, 3446–3449 (2011). 21721053 10.1002/adma.201101345

[b40] Sun G. *et al.* Hybrid Fibers Made of Molybdenum Disulfide, Reduced Graphene Oxide, and Multi-Walled Carbon Nanotubes for Solid-State, Flexible, Asymmetric Supercapacitors. Angew. Chem. Int. Ed. 54, 4651–4656 (2015). 10.1002/anie.201411533 25694387

[b41] Sun G. *et al.* Fabrication of Ultralong Hybrid Microfibers from Nanosheets of Reduced Graphene Oxide and Transition-Metal Dichalcogenides and their Application as Supercapacitors. Angew. Chem. Int. Ed. 53, 12576–12580 (2014). 10.1002/anie.201405325 25130600

[b42] Chmiola J. *et al.* Monolithic Carbide-Derived Carbon Films for Micro-Supercapacitors. Science 328, 480–483 (2010). 20413497 10.1126/science.1184126

[b43] Cao L. *et al.* Direct Laser-Patterned Micro-Supercapacitors from Paintable MoS_2_ Films. Small 9, 2905–2910 (2013). 23589515 10.1002/smll.201203164

[b44] Yang S. *et al.* Bottom-up Approach toward Single-Crystalline VO_2_-Graphene Ribbons as Cathodes for Ultrafast Lithium Storage. Nano Lett. 13, 1596–1601 (2013). 23477543 10.1021/nl400001u

[b45] Chang K. & Chen W. L-Cysteine-Assisted Synthesis of Layered MoS_2_/Graphene Composites with Excellent Electrochemical Performances for Lithium Ion Batteries. ACS Nano 5, 4720–4728 (2011). 21574610 10.1021/nn200659w

