# Supplementary material for: Large-Scale Production of Large-Size Atomically Thin Semiconducting Molybdenum Dichalcogenide Sheets in Water and Its Application for Supercapacitor
Source: Sci Rep. 2016 May 26;6:26660. doi: 10.1038/srep26660 (PMC4881041; doi:10.1038/srep26660)
Supplement: Supplementary Information [file srep26660-s1.doc]

Supplementary Information

Large-Scale Production of Large-Size Atomically Thin Semiconducting Molybdenum Dichalcogenide Sheets in Water and Its Application for Supercapacitor

Yu-Xiang Chen^1^, Chien-Wei Wu^2^, Ting-Yang Kuo ^2^, Yu-Lung Chang^1^, Ming-Hsing Jen^1^, I-Wen Peter Chen^1,*^

^1^ Department of Applied Science, National Taitung University, 369, Sec. 2, University Rd., Taitung City 95092 (Taiwan)

^2^ Department of Chemistry, National Taiwan University, 1, Sec. 4, Roosevelt Road, Taipei, 10617 (Taiwan)

^*^Corresponding. iwchen@nttu.edu.tw


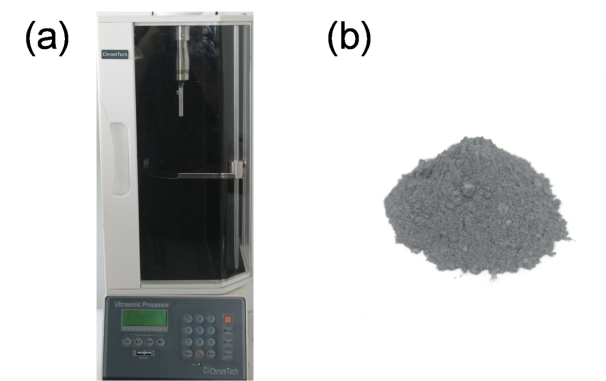


Figure S1. (a) The ultrasonic homogenizer (model: Chromtech UP-500) with an ultrasonic probe and controller. (b) Photograph of the MoS_2_ starting powder.


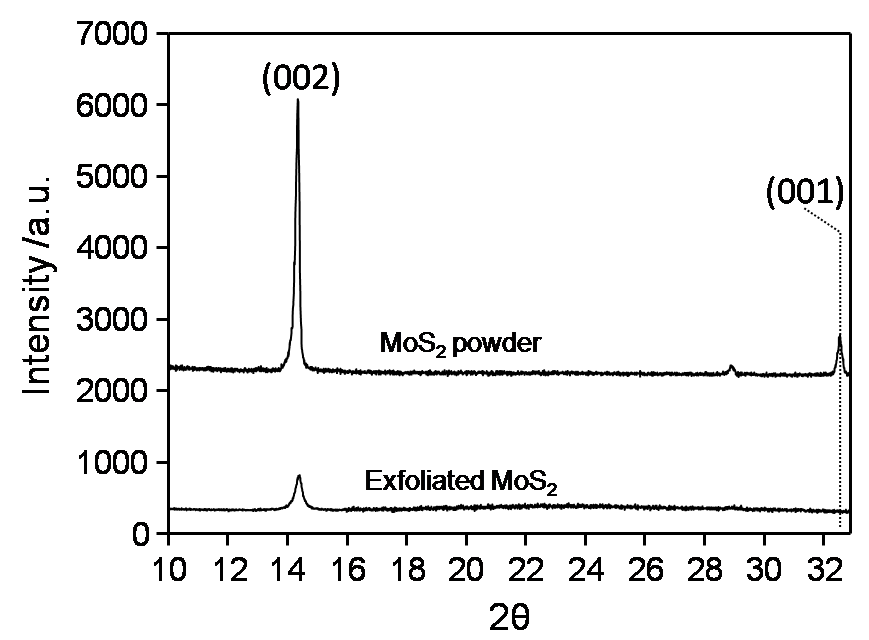


Figure S2. XRD patterns of exfoliated 2H-MoS_2_ sheets and MoS_2_ powder.


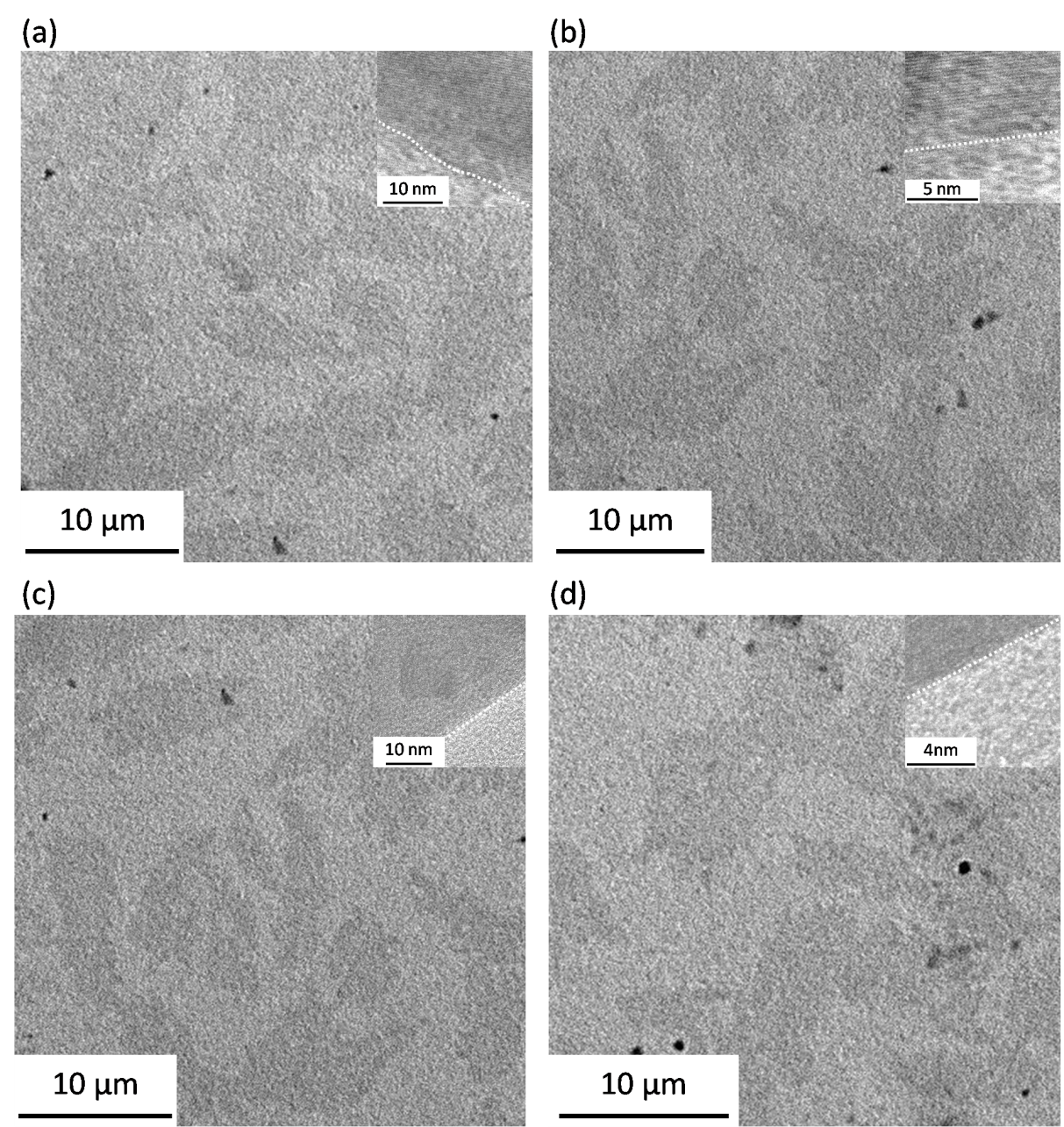


Figure S3. Low-resolution TEM image of large-area 2H-MoS_2_ flakes. Insets: HRTEM images of the edge of the exfoliated 2H-MoS_2_. White dotted line indicates the edge of the selected MoS_2_ sheet.


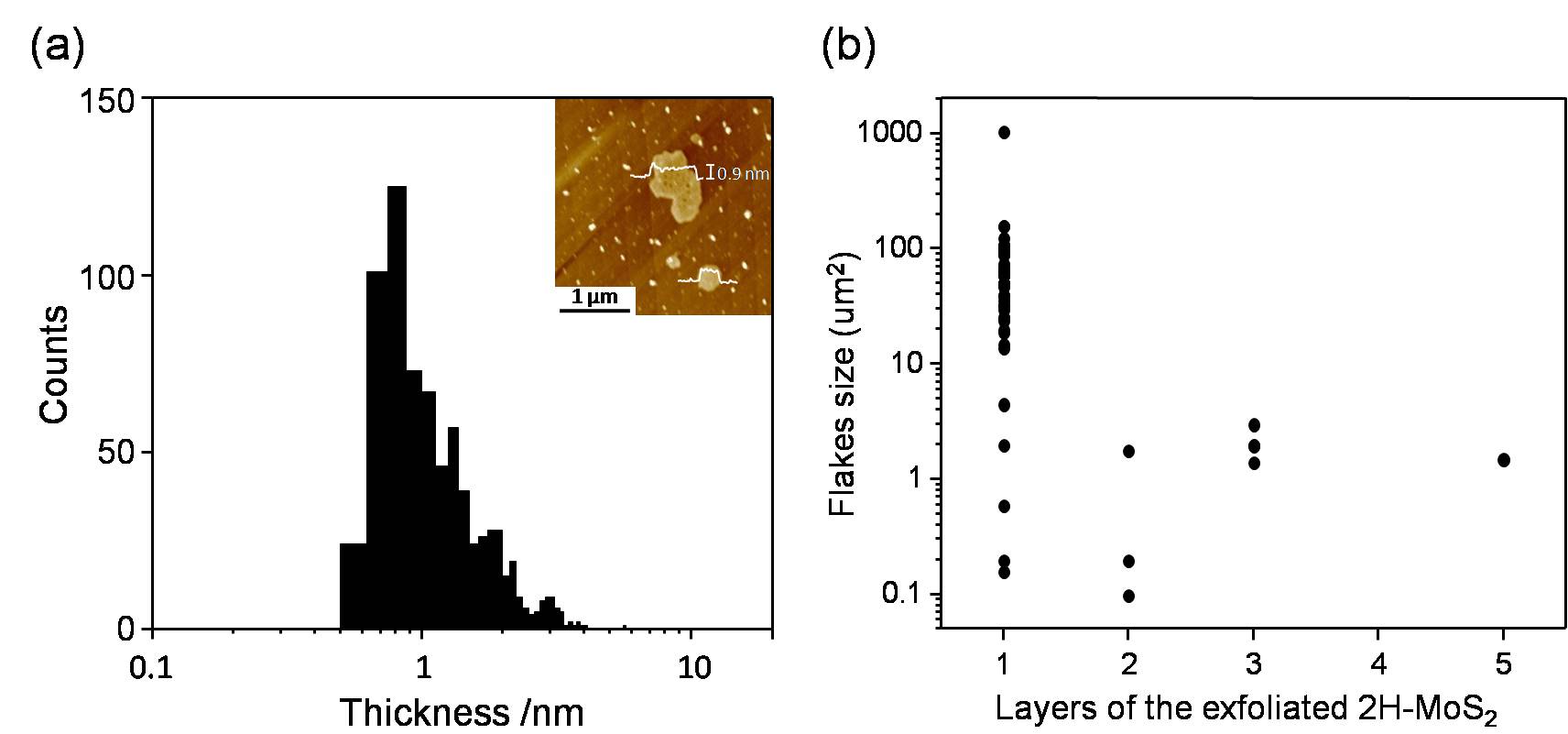


Figure S4. (a) Thickness histogram constructed from 743 exfoliated 2H-MoS_2_ sheets via AFM images. (b) Layer distribution against 2H-MoS2 flake size distributions via TEM images.

Figure S5. Proposed schematic illustration for the exfoliation of the 2H-MoS_2_ powder.


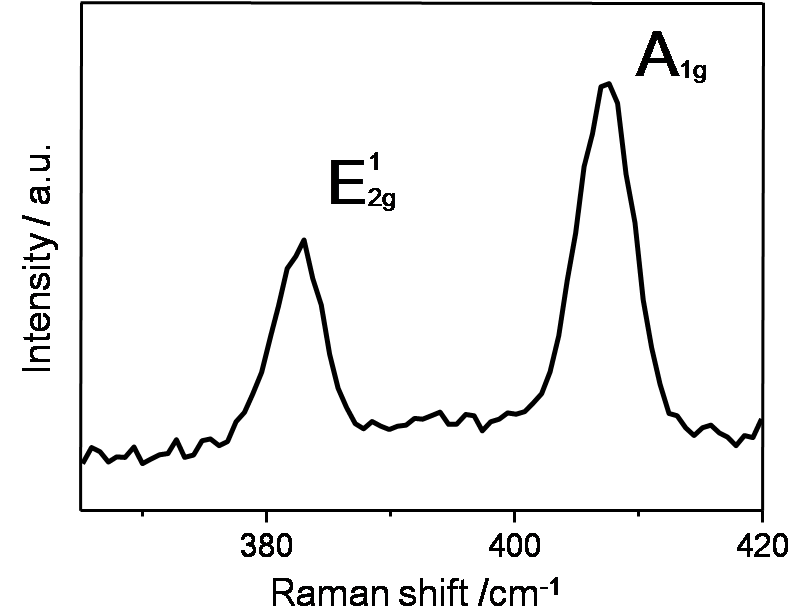


Figure S6. Typical Raman spectrum of Py^+^-assisted exfoliated 2H-MoS_2_ sheets.


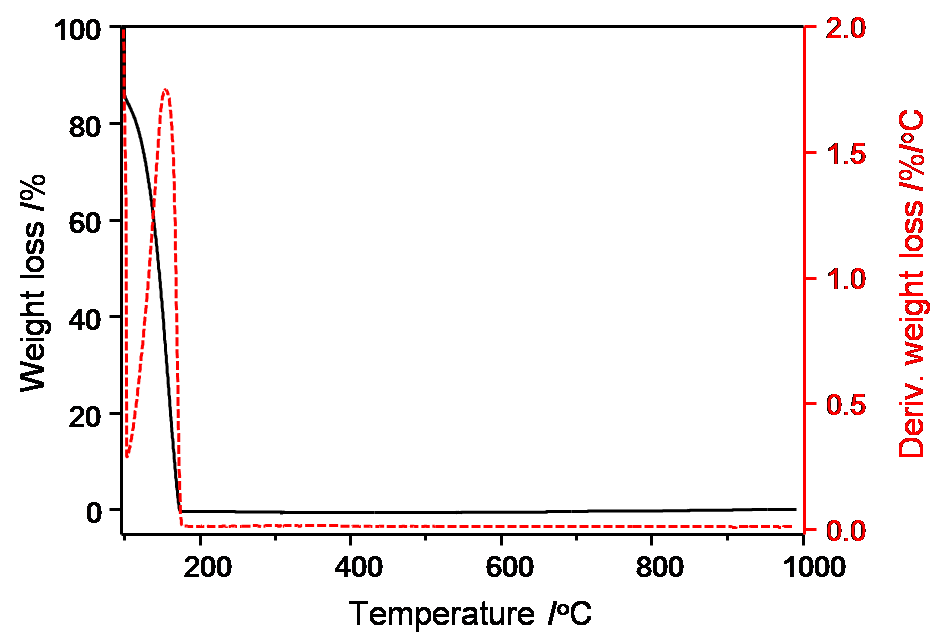


Figure S7. TGA data on the ImH powder.


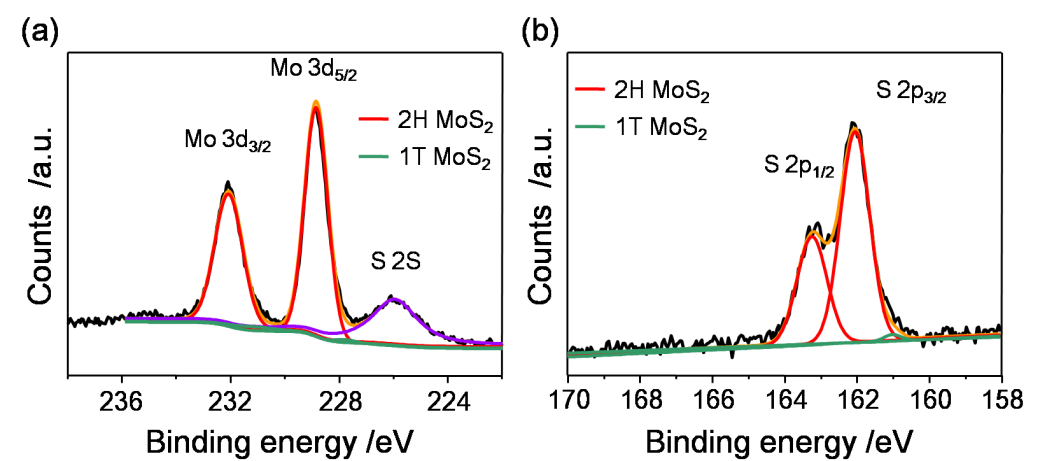


Figure S8. XPS spectra of MoS_2_ annealed at 200 ^o^C. (a) The peak regions of Mo 3d and S 2s core level for the exfoliated 2H-MoS_2_ sheets. (b) The S 2p core level of the XPS spectrum of exfoliated 2H-MoS_2_ sheets.


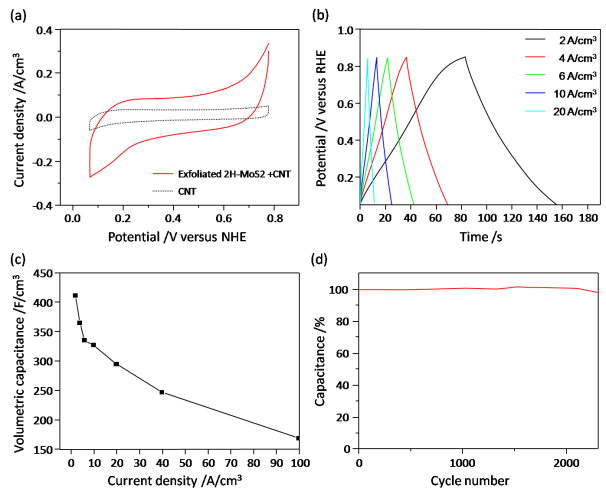


Figure S9. (a) CV curve of supercapacitors based on 2H-MoS_2_/CNTs papers electrode at a scan rate of 1 mV/s. (b) Typical charging/discharging curves with discharging currents density from 2 to 20 A/cm^3^. (c) Current density dependent of 2H-MoS2/CNTs paper-based supercapacitor. (d) Cycling stability of 2H-MoS_2_/CNTs paper based supercapacitor for 2,300 cycles.
